# Ultrafast femtosecond pressure modulation of structure and exciton kinetics in 2D halide perovskites for enhanced light response and stability

**DOI:** 10.1038/s41467-021-25140-2

**Published:** 2021-08-12

**Authors:** Chunpeng Song, Huanrui Yang, Feng Liu, Gary J. Cheng

**Affiliations:** 1 The Institute of Technological Sciences, Wuhan University, Wuhan, China; 2 Brick Nanotechnology Center, Purdue University, West Lafayette, IN USA; 3 School of Industrial Engineering, Purdue University, West Lafayette, IN USA

**Keywords:** Two-dimensional materials, Electronic properties and materials

## Abstract

The carriers’ transportation between layers of two-dimensional (2D) perovskites is inhibited by dielectric confinement. Here, for the first time, we employ a femtosecond laser to introduce ultrafast shock pressure in the range of 0~15.45 GPa to reduce dielectric confinement by modulating the structure and exciton dynamics in a perovskite single crystal (PSCs), e.g. (F-PEA)_2_PbI_4_ (4-fluorophenethylammonium, F-PEA). The density functional theory (DFT) simulation and experimental results show that the inorganic framework distortion results in a bandgap reduction. It was found that the exciton-optical phonon coupling and free excitons (FEs) binding energy are minimized at 2.75 GPa shock pressure due to a reduction in dielectric confinement. The stability testing under various harsh light and humid thermal conditions shows that femtosecond laser shocking improves the stability of (F-PEA)2PbI4 PSCs. Femtosecond laser shock processing provides a new approach for regulating the structure and enhancing halide perovskite properties.

## Abstract (web summary)

Exciton in two-dimensional perovskite is strongly influenced by dielectric confinement of the organic components. Here, the authors employ femtosecond laser to induce ultrashock pressure to investigate how the structural changes and the reduction of dielectric confinement affects exciton behaviour and dynamic.

## Introduction

The 3D organic–inorganic halide perovskite as light-absorbing materials for photovoltaic devices has attracted extensive attention with certified power conversion efficiencies (PCE) exceeding 23% in just over a decade^1–3^. However, perovskite’s long-term stability remains a pressing challenge. 2D perovskite is one type of quantum-well (QW)-like materials representing R_2_A_n−1_B_n_X_3n+1_, where R and A are organic cations, B is a metal cation, X is a halide, and n value is metal cation layers thickness. The n value determines the quantum and the dielectric confinement degree^4^, and the *R* influences the stability^5^ as well as flexibility^6^, which enables the modulation of the performance of 2D perovskite photoelectric to the greatest extent. Therefore, 2D perovskites have many promising applications in light-emitting diodes (LED)^7^, detectors^8^, and lasers^9^ owing to excellent light, thermal, and humid thermal stability compared to their 3D counterparts^10–12^.

The large size of the organic spacer cations (R) for 2D perovskite permits absorption in the visible range significantly lower than that of its 3D counterpart^13,14^. In other words, a large bandgap in 2D perovskites inhibited light utilization at 500–780 nm. Besides, the tremendous exciton binding energy in 2D perovskites is mainly attributed to dielectric confinement^15^. Several hundred meV binding energy is difficult to separate for the free carriers collected by the electrode without an external drive^16^. Regulating the structure of 2D perovskite and exciton transport dynamics is a key to improve its efficiency. Because of the soft lattice nature and the flexibility of sizeable organic spacer cations, the pressure control in the 2D perovskite structure becomes a vital means^17,18^. Currently, most of the research focuses on structure modulation via diamond anvil cell (DAC), while the kinetics of exciton transport after pressure is of little concern^19,20^. Ultrafast laser shocking has a much shorter mechanical interaction time that differs from DAC in maintaining the metastable state of the structure, while the structure after DAC is a reversible process and returns to its original condition after processing, which makes it difficult to control the structure and properties. In addition, the ultrashort energy deposition time limits the thermal energy diffusion away from the laser-shock zone, which is beneficial to reduce the irreversible damage to the material caused by heat. Controlling the plastic deformation generated by local residual compressive stress caused by the pulse laser-shock wave propagating in the material is beneficial to realize the control of local defects^21^. Simultaneously, the laser shocking pressure can be tuned from one to hundreds of gigapascals with selected pulse duration, energy density, and ablator^22,23^. Besides, ultrashort energy deposition time and the adjustability of pulse laser beam size promise to accomplish laser-shock processing in a short time. Thus, femtosecond laser shocking has unparalleled advantages over DAC in large-area controllable processing. The application of the shock pressing with a nanosecond pulse laser has been applied in residual stress regulation and grain size optimization of perovskite thin films^24^, nanolithography of metallic thin films^25^, strain engineering^26^, and patterned transformation^27^. However, the ultrashort energy deposition time and wide ranges of pressure control in femtosecond laser-shock processing will bring many different conditions for structure changes and exciton kinetics in semiconductors and fully utilize the potential advantages of ultrafast shock processing.

In this work, we investigate the structure and exciton kinetics of (F-PEA)_2_PbI_4_ PSCs under femtosecond laser shock in the range of 0–15.45 GPa. To our knowledge, it is the first application of pulsed femtosecond laser shocking in halide perovskites as well as the relationship between crystal structure and exciton kinetics of ultrafast pressure variation in perovskite was studied systematically, which photodetector device performance have been clear as well. First, the 150 meV reduction of the bandgap of (F-PEA)_2_PbI_4_ PSCs after ultrafast pressure rise to 15.45 GPa, appearing a critical pressure at 2.75 GPa. The crystal structure and DFT simulation analysis shows that the change of Pb 6*p* and I 5*p* orbitals overlap caused by the distortion of the inorganic frameworks is the main reason for the band structure change. Besides, the binding energy and the exciton-optical phonon coupling of FEs are weakened to promote exciton separation. The balanced electron/hole effective mass and femtosecond timescale shock wave restrict defects, result in carrier transport and collection enhancement, which boosts photocurrent from 75 to 403 nA in an (F-PEA)_2_PbI_4_ PSCs photodetector. Finally, the stability testing under various harsh light and humid thermal conditions shows that ultrafast pressure improves the stability of (F-PEA)_2_PbI_4_ PSCs by fine-tuning the inorganic frameworks. Therefore, ultrafast laser shocking provides a facile and robust way to modulate the structure and exciton dynamics of perovskites and enhance their properties.

## Results

### Femtosecond laser shocking ultrafast pressure process

**Fig. 1:**
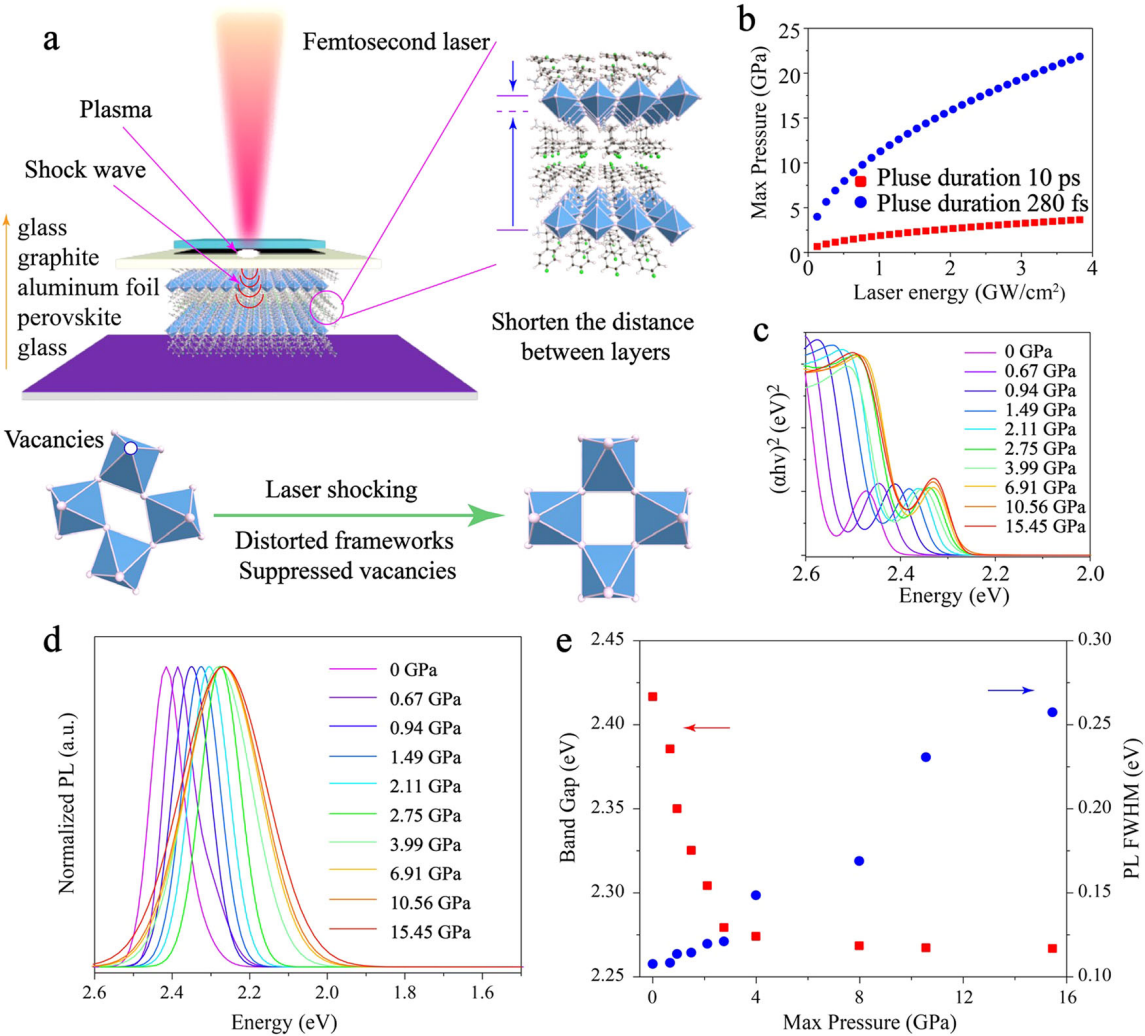
The bandgap modulation of (F-PEA)_2_PbI_4_ PSCs via femtosecond laser shocking. **a** Schematic of the laser-shock deformation process of (F-PEA)_2_PbI_4_ PSCs. **b** The relationship between laser energy density and maximum laser shocking pressure. **c** Tauc curves obtained from ultraviolet-visible absorption spectra reveal bandgap values variation with ultrafast pressure. **d** photoluminescence spectra under ultrafast pressure. **e** According to the Tauc curve, the bandgap decreases with ultrafast pressure, while the full-width at half-maximum (FWHM) of photoluminescence (PL) spectra is the opposite.

Most 2D Perovskites are malleable because of their soft nature lattice and flexible organic cations. The relationship between crystal structure and photoluminescence (PL) emission of 2D perovskite compressive by DAC pressure has received significant attention^17,28^. However, the local plastic deformation resulting from femtosecond laser shocking has more advantages in strain control than elastic deformation induced by DAC pressure processing. The schematic of the femtosecond laser-shock deformation process of 2D perovskite is shown in Fig. 1a. A high energy density femtosecond laser beam was focused onto the graphite surface, generating plasma, inducing a pulse pressure as a result of the plasma recoil momentum. Transparent dielectric glass restricted the hot plasma adiabatic expansion, resulting in an ultrafast shock pressure toward perovskites^29^. Besides, aluminum foil with high thermal conductivity acted as the thermal conduction and isolation layers, avoiding tension stress and pollution of perovskite surfaces caused by direct plasma heating. The 2D perovskites were subjected to femtosecond laser-induced ultrafast pressure perpendicular to the inorganic frameworks, which induces two deformation modes to take place, i.e., the reduction of interlayer distance and distortion of the inorganic frameworks. Ultrafast pressure enhances benzene dipole interaction and π–π conjugate, and promotes carriers’ transport, while the distortion of the inorganic frameworks reduced the long-distance order of 2D perovskite. The regulation of these two competitive processes has been a primary method to modulate the optoelectronic properties and structure of 2D perovskite.

The ultrafast pressure is controlled by laser power density, ablator, and pulse duration^30^. The maximum ultrafast pressure and shock wave velocity were calculated, which increased with the laser energy increasing and decreasing pulse duration (Fig. 1b and calculation). Simultaneously, the shock wave is spherical expansion and penetration depth up to dozens of millimeters^31^. The thickness of (F-PEA)_2_PbI_4_ PSCs has only 15 μm (Supplementary Table 1), which far less than the penetration depth of the shock wave. As a consequence, we ignore the shock wave decay in perovskite. Shock wave induced by femtosecond travels at a speed of thousands of meters per second through the flyer (aluminum foil) to reach the perovskite surface. The femtosecond energy deposition time limits the diffusion of thermal energy away from the laser-shock zone. Meanwhile, due to the rapid cooling of plasma, the duration of the shock wave is approximately equal to the pulsed laser duration. According to Ballard model^32^, the residual compressive stress is positively correlated with the pulse duration, which is the highest at (F-PEA)_2_PbI_4_ PSCs surface. Besides, the vacancy defects on the halide perovskite surface are mainly caused by ion migration, which is suppressed by residual compressive stress^33^. Ion migration occurs on a timescale from seconds to minutes^34^, whose timescale is orders of magnitude longer than the duration of the shock wave. As a result, the compression and decompression processes were completed before the defects were diffused. Femtosecond laser ultrafast shock wave compression and decompression manage the local compressive strain of 2D perovskite^21,35^. Accordingly, ultrashort mechanical interaction time of pulsed laser shocking has incomparable advantages in material structure and performance regulation.

### Ultrafast pressure balance band structure

To investigate the structure and performance of 2D perovskite after femtosecond laser shocking,(F-PEA)_2_PbI_4_ PSCs were fabricated by a cooling solution method^36^. The crystal structure and morphology were characterized by powder X-ray diffraction (PXRD) and field emission scanning electron microscopy (FE-SEM) mapping as well as energy-dispersive X-ray spectroscopy (EDS), as shown in Supplementary Figs. 2–5. The crystal orientation, distribution, and proportion of elements demonstrate that high-quality (F-PEA)_2_PbI_4_ PSCs had been synthesized. Figure 1c illustrated the bandgap variation of (F-PEA)_2_PbI_4_ PSCs under ultrafast shock wave pressure. With the gradual increase of pressure, the perovskite absorption spectrum and photoluminescence (PL) spectrum are redshift. The asymmetric emission spectrum, which was delivered from trapped states at atmospheric pressure, subsequently disappeared with the increased ultrafast pressure (Fig. 1d). Significant Stokes shift cannot be observed, indicating that nonradiative recombination energy loss is tiny with ultrafast pressure. By comparing the bandgap and PL full-width at half-maximum (FWHM), we found that the bandgap reduced by 150 meV as pressure increase from 0 to 2.75 GPa, while on the contrary, the PL FWHM increased from 0.10 to 0.12 eV (Fig. 1e). Further increase of pressure from 2.75 GPa on (F-PEA)_2_PbI_4_ PSCs would cause irreversible plastic deformation, similar to the amorphous of other perovskites under high pressure^37,38^. We examined the bandgap change over time to determine the (F-PEA)_2_PbI_4_ PSCs structure’s stability (Supplementary Fig. 7). We found that the bandgap remained stable for 144 h after applying the femtosecond shock, and the absorption bandgap could not recover. Therefore, we speculate that the structural distortion of perovskite after femtosecond laser shock can remain stable for at least 144 h. The ultrafast femtosecond pressure modulation was also applied on (PEA)_2_PbI_4_ PSCs. As shown in an ultraviolet-visible (UV-Vis) absorption spectrum (Supplementary Fig. 8), a similar behavior with (F-PEA)_2_PbI_4_ PSCs was discovered, indicating femtosecond laser-shock-induced structural modulation can be extended to other similar materials.

**Fig. 2:**
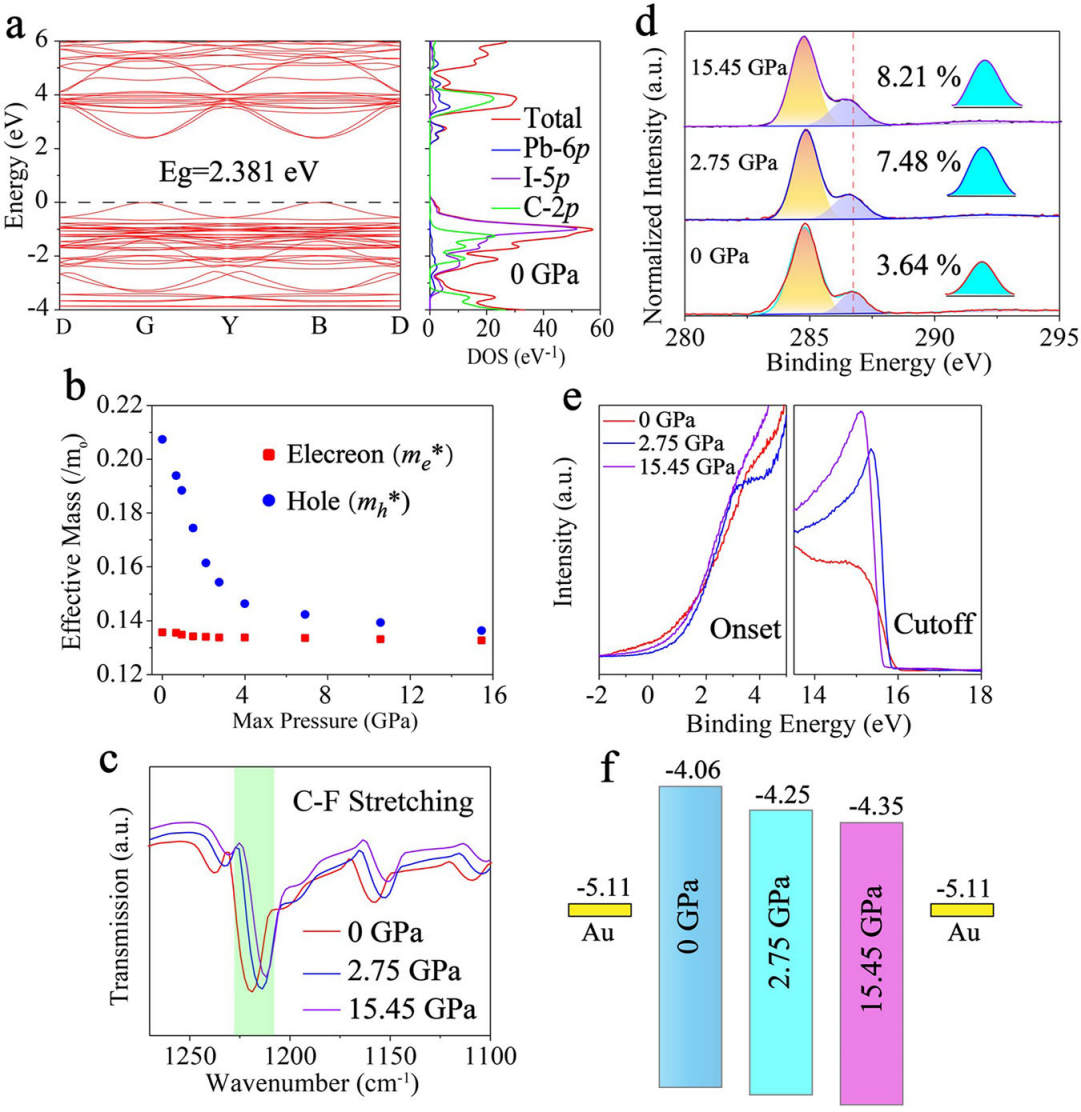
Ultrafast pressure balance band structure of (F-PEA)_2_PbI_4_ PSCs. **a** Density functional theory simulated the band structure and density of states (DOS) without ultrafast pressure, **b** Simulated perovskite effective mass as a function of ultrafast pressure. **c** Fourier transform infrared spectra of (F-PEA)_2_PbI_4_ PSCs. **d** X-ray photoelectron spectroscopy spectra at three representative ultrafast pressures of (F-PEA)_2_PbI_4_ PSCs. **e** Ultraviolet photoelectron spectroscopy spectra of onset and cutoff for (F-PEA)_2_PbI_4_ PSCs. **f** Schematic diagram of band alignment of (F-PEA)_2_PbI_4_ PSCs relative to Au under ultrafast pressure.

The band structure balance between the layers reduces the carrier transmission barrier and the carrier accumulation at the interface, which is beneficial to both carrier transmission and collection. The band structure and density of states (DOS) for the DFT simulated (F-PEA)_2_PbI_4_ PSCs after ultrafast pressure were summarized in Fig. 2a and Supplementary Fig. 18. Partial density of states (PDOS) revealed that the conduction band minima (CBM) and valence band maxima (VBM) of (F-PEA)_2_PbI_4_ PSCs came from Pb 6*p* and I 5*p* orbitals, respectively. Nevertheless, the organic cation was not able to make a direct contribution. The (F-PEA)_2_PbI_4_ PSCs is a direct bandgap semiconductor with a calculated bandgap of 2.381 eV. The bandgap decreases rapidly to 2.215 eV at 2.75 GPa and continues decreasing at a slower rate under shock pressure from 2.75 to 15.45 GPa (Supplementary Fig. 19), which matched the experimental results very well. The effective mass could be evaluated by ${{m}_{e,h}}^{\ast }={{\hslash }}/[{\partial }^{2}\varepsilon ({{{\bf{k}}}})/\partial {{{{\bf{k}}}}}^{2}]$ where ε(k) is the eigenvalue of the wave vector k along the transport direction, and ℏ is Planck’s constant. The effective mass of electrons (*m*_e_*) and holes (*m*_h_*) with pressure were summarized in Fig. 2b. The *m*_h_* decreased with the pressure and finally approached *m*_e_* at 15.45 GPa^34^. The balanced electron/hole effective mass promotes carriers’ transport and collection, owing to local residual compressive stress regulation as a result of ultrafast compress and decompress via femtosecond laser shocking. Moreover, we have studied the molecular interaction of perovskite in the Fourier transform infrared (FTIR) spectrum. With femtosecond laser shock, C-F stretching and benzene vibration frequencies are enhanced, as shown in Fig. 2c and Supplementary Fig. 20. X-ray photoelectron spectroscopy (XPS) of (F-PEA)_2_PbI_4_ PSCs at atmospheric pressure shown that three C 1 *s* peaks: C–C (284.79 eV), C–N (286.72 eV), and π–π (292.24 eV)^3^ (Fig. 2d). The increased pressure weakens the C–N binding energy, while the C–C remains unchanged. The N 1 *s* peak was blueshift and the Pb 4 *f* peak was redshift, as shown in Supplementary Fig. 21. This contrary change confirmed that the organic cation indirectly affecting the band structure through the N–H$\cdots$I hydrogen bonding^39^. The relative peak area of π–π interaction as a function of pressure announced that dipole interaction between layers raised, promoting carriers to pass through organic layers^40^. The work function and VBM were obtained by ultraviolet photoelectron spectroscopy (UPS)^41^, as delineated in Fig. 2e. The energy between the VBM and the Fermi level as well as the work function enlarged with ultrafast shock pressure. The CBM and VBM of (F-PEA)_2_PbI_4_ PSCs shifted down to the deep energy level (Fig. 2f). Also, the CBM was gradually aligned with the Au, reducing electronic potential barriers between (F-PEA)_2_PbI_4_ PSCs.

### The source of band structure regulation

**Fig. 3:**
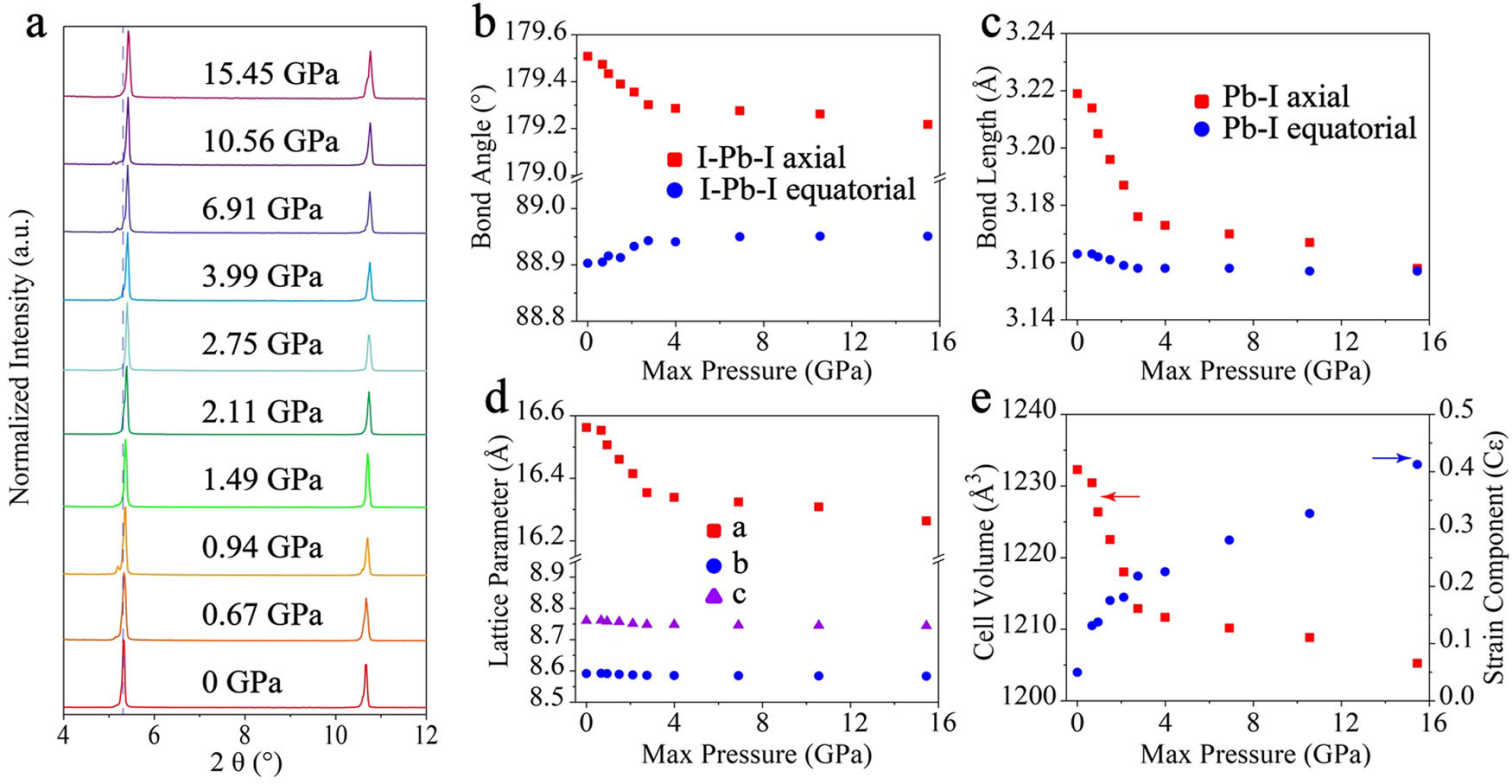
Ultrafast pressure regulation lattice of (F-PEA)_2_PbI_4_ PSCs. **a** With the increase of ultrafast pressure, the powder X-ray diffraction spectrum gradually redshifts for (F-PEA)_2_PbI_4_ PSCs. **b** Ultrafast pressure-dependent I–Pb–I bond angles and **c** Pb–I bond lengths. Obviously, the inorganic frameworks distorted with pressure can be observed. **d** Unit cell parameters of (F-PEA)_2_PbI_4_ PSCs as a function of pressure. **e** Unit cell volumes evolution and Williamson–Hall strain component of (F-PEA)_2_PbI_4_ PSCs with increasing ultrafast pressure.

The crystal structure of the perovskite directly affects its properties and the management of the (F-PEA)_2_PbI_4_ PSCs lattice has become an important method to regulate its photoelectric performance. Crystallographic data for (F-PEA)_2_PbI_4_ PSCs were summarized in Supplementary Table 1, in which (F-PEA)_2_PbI_4_ PSCs are monoclinic systems with P2_1_/c(#14) group^42^. Figure 3a shows the PXRD pattern with pressure, the (001) peak of (F-PEA)_2_PbI_4_ PSCs redshifts as the pressure. There is no new crystal peak within the ultrafast pressure range of our experiment, indicating that the irreversible phase transition does not occur after ultrafast compression and decompression. Figure 3b, c summarizes the relationship between bond Angle and bond length with pressure. The bond angle and bond length change from 0 to 2.75 GPa significantly exceeded that from 2.75 to 15.45 GPa. Moreover, the axial shortening of the Pb–I bond length beyond that of the equatorial, consistent with the I–Pb–I angle. The a axis of (F-PEA)_2_PbI_4_ PSCs unit cell decreased by about 0.3 Å, while the *b* and *c* axes remain unchanged, indicating that the pressure is mainly in the axial compression (Fig. 2d). The reduction of unit cell lattice constants was more significant than the bond length, indicating the organic cation is more compressible than the inorganic frameworks. The unit cell volume also downturn appropriately, and the inflection at 2.75 GPa marked the compression limit of (F-PEA)_2_PbI_4_ PSCs. Williamson–Hall plot was used to estimate the stress in the lattice^43,44^. The slope of the plot represented the strain constant (Cε), but it was not to be taken strictly, which performed the diversity of strain to some extent (Supplementary Fig. 22). *Cε* as a function of max pressure was demonstrated in Fig. 2e. The stress accumulates continuously in the lattices and is altered at 2.75 GPa as well. High lattice strain under 15.45 GPa weakens the long-range order of (F-PEA)_2_PbI_4_ PSCs, conformed with PL peak broadening.

### Modulation of exciton kinetics and defects

**Fig. 4:**
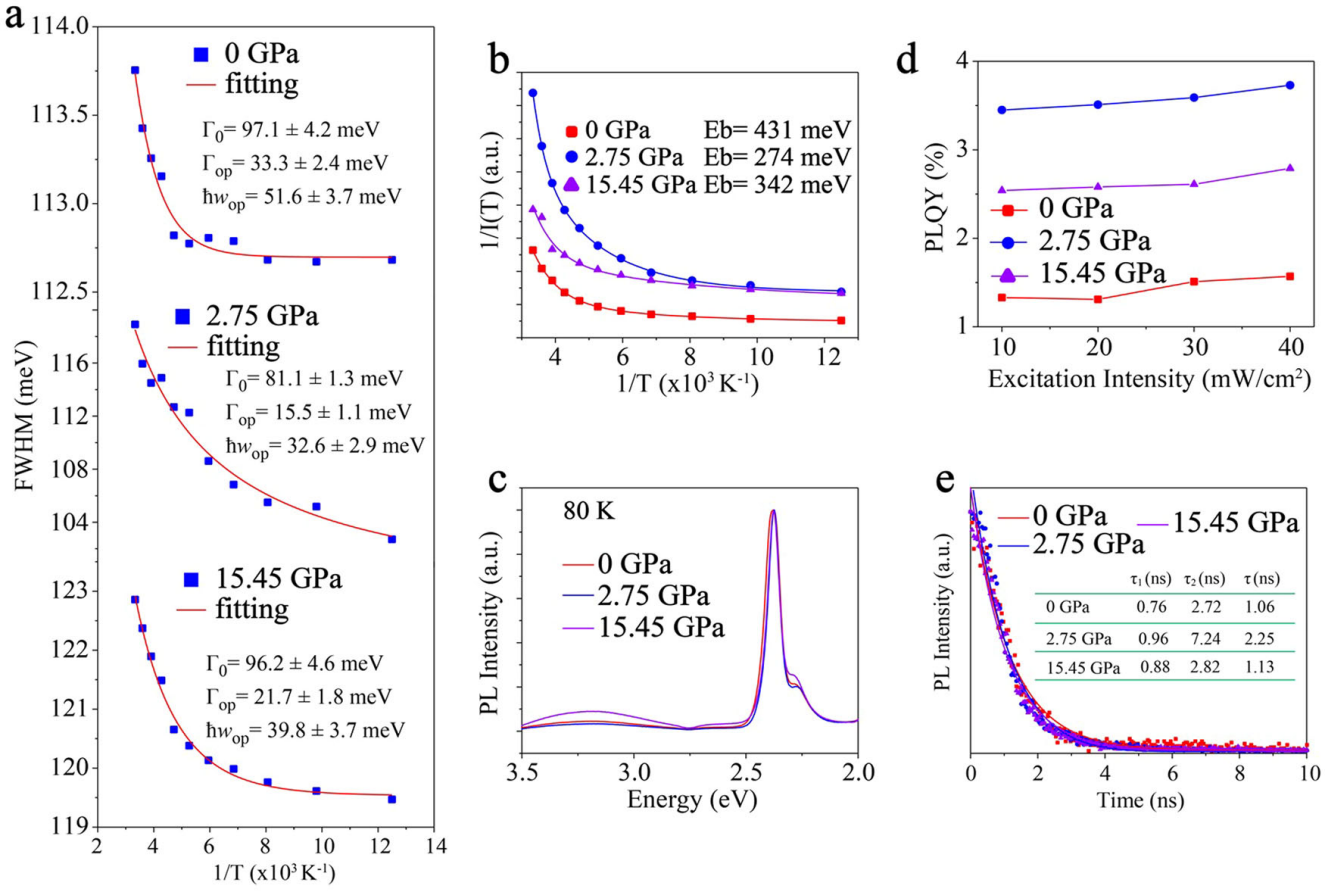
Ultrafast pressure modulation exciton kinetics and defects of (F-PEA)_2_PbI_4_ PSCs. **a** Temperature-dependent photoluminescence (TRPL) full-width at half-maximum (FWHM) of (F-PEA)_2_PbI_4_ PSCs. **b** Free excitons binding energy for (F-PEA)2PbI4 PSCs by fitting temperature-dependent photoluminescence spectra. **c** Photoluminescence spectra of (F-PEA)_2_PbI_4_ PSCs with ultrafast pressure at 80 K. **d** Photoluminescence quantum yields (PLQY) in (F-PEA)_2_PbI_4_ PSCs as a function of femtosecond laser shock for several light-excitation intensities. **e** Carriers lifetime of (F-PEA)_2_PbI_4_ PSCs obtained from TRPL.

The separation of photogeneration excitons is the key to improve the performance of 2D perovskite optoelectronic devices. The exciton binding energy of 2D perovskites is in the range of 100–500 meV, far beyond their 3D counterparts^45,46^. The exciton properties of (F-PEA)_2_PbI_4_ PSCs with ultrafast pressure were investigated in detail by temperature-dependent PL spectra, as depicted in Fig. 4 and Supplementary Fig. 23. The peaks at 1.94 eV and 2.37 eV attribute to shallow trap excitons and FEs, respectively^16,17,47^. Temperature-dependent PL peak width broadening of FEs was fitted with the Boson model:^48^

1 $${\varGamma}(T)={\varGamma}_{0}+\frac{{\varGamma}_{op}}{\exp(\hslash{w}_{op}/{k}_{{{{\rm{B}}}}})-1}$$

Where *Γ*_*0*_ represents the inhomogeneous broadening, *Γ*_*op*_ is the exciton-optical phonon contribution and *ħw*_*op*_ the optical phonon energy. Compared with (F-PEA)_2_PbI_4_ PSCs at atmospheric pressure, the 2.75 GPa femtosecond laser shocking resulted in inhomogeneous broadening and exciton-optical phonon as well as optical phonon energy, decreased to 81.1 ± 1.3, 15.5 ± 1.1, and 32.6 ± 2.9 meV, respectively (Fig. 4a). The fundamental reason is that the enhanced dipole interaction and the reduced dielectric confinement^49^. With the highest ultrafast pressure of 15.45 GPa, the strain accumulated with the disordered electronic structure in (F-PEA)_2_PbI_4_ PSCs, leading to optical phonon scattering enhancement. Inhomogeneous broadening, exciton-optical phonon, and optical phonon energy had been up to 96.2 ± 4.6, 21.7 ± 1.8, and 39.8 ± 3.7 meV, respectively. Their value is still below that at 0 GPa, owing to a reduction in dielectric confinement caused by the π–π enhancement. The temperature-dependent FEs binding energy (*E*_B_) were appraised with the formula^50^:

2 $$I\left(T\right)=\frac{{I}_{0}}{1+A\,{{\exp}} \,(-{E}_{{{{\rm{B}}}}}/{k}_{{{{\rm{B}}}}}T)}$$

in which *I*_0_ and *k*_B_ are intensity at 0 K and Boltzmann constant. The *E*_B_ of (F-PEA)_2_PbI_4_ PSCs at 0 GPa was 431 meV, which was down to 274 meV at 2.75 GPa and up to 342 meV at 15.45 GPa (Fig. 4b). Competition between the disorder of electronic structure and dipole interaction resulted in the minimum *E*_B_ at 2.75 GPa.

We extracted PL spectra at 80 K to investigate shallow trap excitons (at 1.94 eV), as depicted in Fig. 4c. Compared to the shallow trap excitons of (F-PEA)_2_PbI_4_ PSCs at 0 GPa, the value slightly decreased at 2.75 GPa, while it significantly increases at 15.45 GPa, indicating that the defects in perovskite reduce after proper pressure. Besides, pressure changed the energy transfer barrier of FEs and shallow trap excitons, also the inducer of shallow trap excitons diversity^51^. An apparent exciton emission (3.21 eV) peak appeared at 80 K and disappeared at 168 K only after the ultrafast pressure of 15.45 GPa, since exciton-phonon scattering enhances exciton restriction and benzene dipole interaction. Photoluminescence quantum yields (PLQY) for several excitation intensities were used to determine the regulation of (F-PEA)_2_PbI_4_ PSCs by femtosecond laser shock (Fig. 4d). Under air pressure, 1.3% PLQY was obtained, which was much the same as that in previous reports of 2D perovskite^12^. With increasing pressure, PLQY was risen to 3.5% at 2.75 GPa, indicating reduced nonradiative losses. PLQY decreased to 2.6% at 15.45 GPa, resulting from excessive pressure destroying the long-range order of perovskite, which is consistent with PL broadening. The carrier’s lifetime was obtained by fitting the time-resolved photoluminescence (TRPL) spectrum with the 2-constant exponential decay function^24^. We characterized the intensity dependence TRPL and found that the lifetime reached the minimum at the excitation intensity of 300 mW/cm^2^, as shown in Supplementary Fig. 24 and Supplementary Table 6–8. Therefore, TRPL with an excitation intensity of 300 mW/cm^2^ was used to demonstrate exciton quenching lifetime (*τ*_1_) and radiation recombination lifetime (*τ*_2_). The exciton-exciton annihilation lifetime of (F-PEA)_2_PbI_4_ PSCs at 2.75 GPa was just a little bit bigger than those at 0 GPa and 15.45 GPa, respectively, while radiative recombination lifetime was near 2.5 times. The average lifetime (*τ*) was improved by 212%, due to the reduced exciton binding energy and defect density. Also, band structure balance and π–π enhancement of (F-PEA)_2_PbI_4_ PSCs were essential factors in carrier’s lifetime.

### Ultrafast pressure enhanced light response and robust structure

**Fig. 5:**
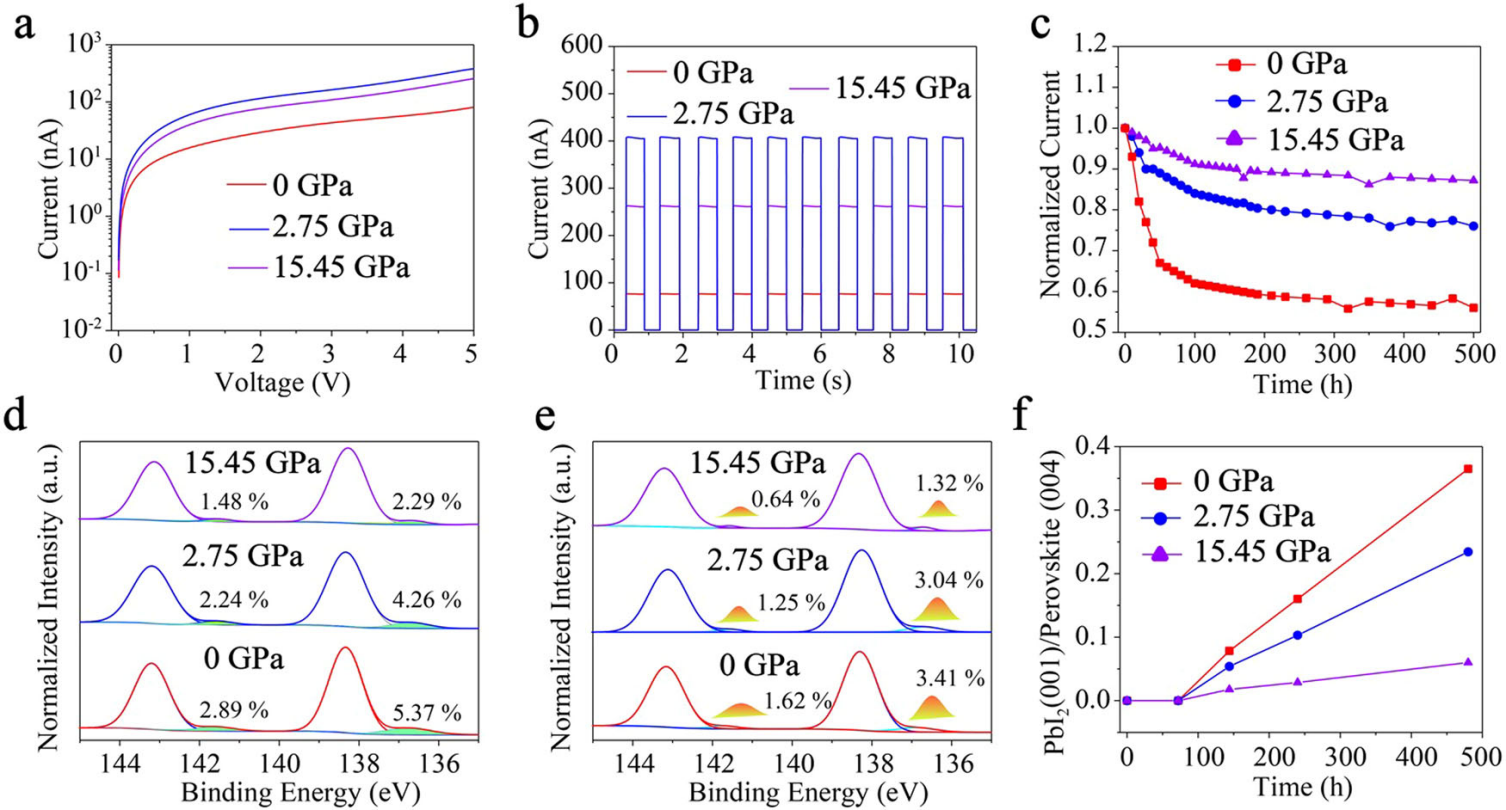
Ultrafast pressure enhanced light response and robust structure for (F-PEA)_2_PbI_4_ PSCs. **a** Bias-dependent photocurrent of the (F-PEA)_2_PbI_4_ PSCs under ultrafast pressure. **b** The photoswitch of (F-PEA)_2_PbI_4_ PSCs photodetector for 5 V bias. **c** Operational stability of nonencapsulated (F-PEA)_2_PbI_4_ PSCs photodetectors, which were fabricated and tested in a room temperature and 85% (±5%) relative humidity ambient atmosphere. Pb binding energy obtained from X-ray photoelectron spectroscopy to assessed **d** light stability (a sunlight input for 10 h) and **e** humid thermal stability (85°C, RH = 85%, 10 h) of (F-PEA)_2_PbI_4_ PSCs. **f** Ratio of the PbI_2_ (2θ = 12.6° referred to <100>) and perovskite (2θ = 10.8° referred to <004>) main peaks over 500 h.

The structure and exciton dynamics of 2D perovskite directly affect its performance. Here, the photoelectric performance of (F-PEA)_2_PbI_4_ PSCs photodetector was characterized by Au/ (F-PEA)_2_PbI_4_ PSCs /Au structure. Photocurrent measurement was carried out on the (F-PEA)_2_PbI_4_ PSCs photodetector with an effective area of 5 × 10^−2^ mm^2^ under 355 nm laser radiation (Intensity, 0.16 mW/cm^2^). Figure 5a shows the current–voltage (I–V) curves of the devices made at various ultrafast pressure. Photocurrent made on 0 GPa and 15.45 GPa with 5 V bias at the same laser radiation was measured to be 75 nA and 257 nA, while its 403 nA at 2.75 GPa, beyond 537% (Fig. 5b). The photocurrent enhancement is attributed to the much reduced FEs binding energy and suppressed exciton-phonon coupling.

Stability is a prerequisite for the practical application of perovskite. we performed device stability measurements under operational conditions for (F-PEA)_2_PbI_4_ PSCs photodetectors. As shown in Fig. 5c, the photocurrent of the perovskite photodetector without pressure decreased rapidly to 67% of the initial photocurrent within 50 h, and finally remained at 56% of the initial photocurrent after 500 h. By comparison, perovskite was applied at ultrafast pressure of 2.75 GPa and 15.45 GPa, maintaining 76% and 87% of the initial photocurrent after 500 h, respectively. XRD and XPS were characterized by the light and humid thermal stability of perovskite. The phase transition and decomposition of (F-PEA)_2_PbI_4_ PSCs were analyzed by differential scanning calorimetry (DSC) under the nitrogen atmosphere^52^ (Supplementary Fig. 26). The fusion temperature of (F-PEA)_2_PbI_4_ PSCs was found to be 518 °C with an increase in enthalpy change under ultrafast pressure. The light stability of (F-PEA)_2_PbI_4_ PSCs increases with the ultrafast pressure under a sunlight input for 10 h, as explained by the Pb^0^/(Pb^0^+Pb^2+^) increment (Fig. 5d)^53^. Figure 5e demonstrates the humid thermal stability of (F-PEA)_2_PbI_4_ PSCs over 10 h with relative humidity (RH) 85% and temperature 85 °C. The Pb^0^/(Pb^0^ + Pb^2+^) was 5.03% without shock treatment, compared to 1.96% at 15.45 GPa shock pressure. Figure 5f and Supplementary Fig. 27 show the air stability of (F-PEA)_2_PbI_4_ PSCs, which are measured at room temperature and 85% (±5%) relative humidity. We found that the crystallization peak of lead iodide (001) appeared at 144 h. The perovskite without femtosecond laser shock decomposed rapidly, while the perovskite decomposed significantly slower after applying ultrafast pressure. We attribute the stability improvement of (F-PEA)_2_PbI_4_ PSCs results from the optimization of lattice and band structure.

## Discussion

In general, we introduce a pulsed laser to shock(F-PEA)_2_PbI_4_ PSCs for the first time and studied its structure and exciton dynamics in detail. The bandgap decreases by 150 meV and remains stable with femtosecond laser shocking. DFT simulation and experimental results show that the reason for the decrease of the bandgap is the distortion of the inorganic frameworks. Through dipole interaction, the organic cation can distort the inorganic frameworks, and N–H$\cdots$I hydrogen bonding indirectly affecting the band structure. By fitting the temperature-dependent PL spectrum, exciton-optical phonon coupling and optical phonon energy are 15.5 ± 1.1 meV and 32.6 ± 2.9 meV at 2.75 GPa. The FEs binding energy is 274 meV at 2.75 GPa, much smaller than that at 0 GPa, owing to the π–π enhancement and dielectric confinement reduction in (F-PEA)_2_PbI_4_ PSCs. The hole effective mass decreases while the electron effective mass is unchanged. The balanced electron/hole mass, as well as defect reduction, promote the carrier transport and collection, owing to local residual compressive stress regulation as a result of ultrafast compress and decompress via femtosecond laser shocking. The photocurrent increasing from 75 to 403 nA of (F-PEA)_2_PbI_4_ PSCs photodetector, owing to carries lifetime increment and suppressed exciton-phonon coupling. More importantly, ultrafast laser shocking is shown to be a feasible method to modulate the structure and physical properties of perovskite, promising to improve electronic and optoelectronic performances of hybrid organic–inorganic halide perovskites and providing a critical solution to the stability as well.

## Methods

### Materials

4-fluorophenethylammonium iodide (F-PEAI, purity > 99%) purchased from Xi’an Polymer Light Technology Corp. Lead(II) oxide (PbO, purity > 99.999%), Hydriodic acid (HI, 57 wt.% in water), Hypophosphorous acid solution (H_3_PO_2_, 50 wt.% in water), and Hexane (purity > 95%) purchased from Sigma–Aldrich. All materials are used as received.

### (F-PEA)_2_PbI_4_ and (PEA)_2_PbI_4_ single-crystal growth

To fabricate (F-PEA)_2_PbI_4_ perovskite single crystal (PSCs), 44.6 mg PbO (0.2 mmol) and 106.8 mg F-PEAI (0.4 mmol) dissolved in HI/ H_3_PO_2_ (4 ml for HI and 0.8 ml for H_3_PO_2_) mix solution. Bring to a boil in an oil bath until completely dissolved into a bright yellow transparent solution. The solution was cooled to 125 °C for 15 min to allow (F-PEA)_2_PbI_4_ PSCs to crystal completely. After being naturally cooled to room temperature, the yellow (F-PEA)_2_PbI_4_ PSCs were filtered and washed with hexane. The (PEA)_2_PbI_4_ single crystals were grown with the same protocol.

### Laser shocking (F-PEA)_2_PbI_4_ PSCs

A Diode-Pumped Femtosecond Industrial Laser (1064 nm wavelength, repetition rate 7.5–375 kHz and pulse duration of 280 fs–10 ps) was used as a laser energy source. A glass slide was used as a confining media because of its high shock impedance. A sacrificial coating was deposited by spraying a graphite ablator above the 10 μm aluminum foil. The graphite layer was instantaneously ablated by a highly focused femtosecond laser (beam size 7–100 μm), which created a shock wave that exerted enormous pressure on (F-PEA)_2_PbI_4_ PSCs and changed its lattice structure. The femtosecond laser scans are performed at a speed of 50 mm/s, with a pulse energy of 0–80 uJ.

### Material characterization

The morphology and energy-dispersive X-ray spectroscopy (EDS) of (F-PEA)_2_PbI_4_ PSCs were obtained by field emission scanning electron microscopy (FE-SEM, Zeiss SIGMA). Atomic force microscope (AFM, SPA 400BRUKER Dimension Edge) was used to perform the perovskite morphology. PE Lambd 950 was used to perform the ultraviolet-visible absorption (UV-Vis) of a single crystal to obtain the optical bandgap of the film. Steady-state photoluminescence and temperature-dependent photoluminescence of perovskite single crystals were characterized by FLS 980 for laser excitation at 315 nm. NICOLET 5700 Fourier transform infrared (FTIR) Spectrometer was used to characterization perovskite Molecular vibrations. Time-resolved photoluminescence (TRPL) data of single-crystal perovskite were collected by FLS1000 using 340 nm laser excitation. Photoluminescence quantum yield (PLQY) of the (F-PEA)_2_PbI_4_ PSCs were measured using an integrating sphere in the air under ambient conditions, and a laser with wavelength 340 nm was used as the excitation source. The crystal structure of (F-PEA)_2_PbI_4_ PSCs was obtained by Bruker D8 Venture. X-ray photoelectron spectroscopy (XPS) and ultraviolet photoelectron spectroscopy (UPS) data were measured by Thermo Fisher Scientific ESCALAB250Xi. Thermogravimetric analysis (TGA) and differential scanning calorimetry (DSC) of 2D single-crystal perovskite have an important application in thermal stability analysis of perovskite by using Mettler-Toledo TGA2 and DSC3, respectively. The thickness was measured using a surface profilometer (KLA-Tencor D-120). Photocurrent measurement was carried out on the (F-PEA)_2_PbI_4_ PSCs photodetector with the structure of Au/(F-PEA)_2_PbI_4_/Au, which was measured by Keysight B2901A oscilloscope under 355 nm laser radiation (Power density, 0.16 mW/cm^2^). The Au-wire with a thickness of 200 nm, a length of 500 μm and 20 μm in the gap, was used as the electrode with an effective area of 5 × 10^−2^ mm^2^.

### Density functional theory simulation

The first-principles calculations were performed using the plane-wave pseudopotential method within the frameworks of DFT as implemented in the Vienna Ab initio Simulation Package^54^. The generalized gradient approximation Perdew–Burke–Ernzerhof (PBE) exchange-correlation functional was used^41^. Band structure of (F-PEA)_2_PbI_4_ PSCs was carried out along the symmetrical point D (−0.5, 0, 0.5), G(0, 0, 0), Y(0, 0.5, 0), B(−0.5, 0, 0), and D. A 5 × 5 × 1 Monkhorst-Pack k-point mesh for electronic Brillouin zone integration of 2D perovskites single crystal with cutoff energy 500 eV.

### Calculations

The max pressure of laser shocking was calculated by^30^:

3 $$P\left({\rm T}\right)={[\frac{{mI}{{{\rm{\alpha }}}}}{2T\left({{{\rm{\alpha }}}}+1\right)}]}^{1/2}$$

Where *T* is the pulse duration of the femtosecond laser, *I* represent laser energy density of one pule (in GW/cm^2^), α is a parameter of the reflected laser capability of the ablation layer (*α* = 0.1 in graphite). The corresponding laser ablation mass is expressed in terms of *m*, which is determined by the mass change before and after the femtosecond laser. Simultaneously, we used a slightly excessive amount of graphite to avoid aluminum foil interference in the mass calculation.

We quantified the straining of (F-PEA)_2_PbI_4_ PSCs under laser shocking pressure by fitting the PXRD peak broadening, as follow:^44^

4 $$\Delta {d}_{\varepsilon }=\sqrt{\Delta {{d}_{{{{{\mathrm{obs}}}}}}}^{2}-\Delta {{d}_{{{{{\mathrm{int}}}}}}}^{2}}={C}_{\varepsilon }d$$

where Δ*d*_obs_^2^ is the difference in d-spacing at full-width at half-maximum (FWHM) for a given plane. Δ*d*_int_^2^contribution from the instrument and *d* is interplanar spacing.

TRPL spectra of (F-PEA)_2_PbI_4_ PSCs floating on the glass were fitted by 2-constant exponential decay function:^55^

5 $$f\left(t\right)={{{{\rm{A}}}}}_{1}{{\exp }}\left(-\frac{t}{{\tau }_{1}}\right)+{{{{\rm{A}}}}}_{2}{{\exp }}\left(-\frac{t}{{\tau }_{2}}\right)+{{{\rm{B}}}}$$

where A is the decay amplitude, *τ*_1_ and *τ*_2_ are carrier lifetimes, corresponding to extraction time and radiative recombination lifetime.

## Supplementary information

Supplementary Information

## Data Availability

The data that support the findings of this study are available on reasonable request from the corresponding author.
