# Supplementary material for: Ultrafast femtosecond pressure modulation of structure and exciton kinetics in 2D halide perovskites for enhanced light response and stability
Source: Nat Commun. 2021 Aug 12;12:4879. doi: 10.1038/s41467-021-25140-2 (PMC8361179; doi:10.1038/s41467-021-25140-2)
Supplement: Supplementary file 1 — Supplementary Information [file 41467_2021_25140_MOESM1_ESM.pdf]

Supplementary Information for:

Ultrafast Femtosecond Pressure Modulation of Structure and Exciton Kinetics in 2D Halide Perovskites for Enhanced Light Response and Stability

Chunpeng Song[1], Huanrui Yang[1], Feng Liu[1], Gary J. Cheng[1,2,3*]

[1]The Institute of Technological Sciences, Wuhan University, Wuhan 430072, China

[2]Brick Nanotechnology Center, Purdue University, West Lafayette, IN, USA

[3]School of Industrial Engineering, Purdue University, West Lafayette, IN, USA

[*]Corresponding authors: gjcheng@purdue.edu.

Supplementary Figures

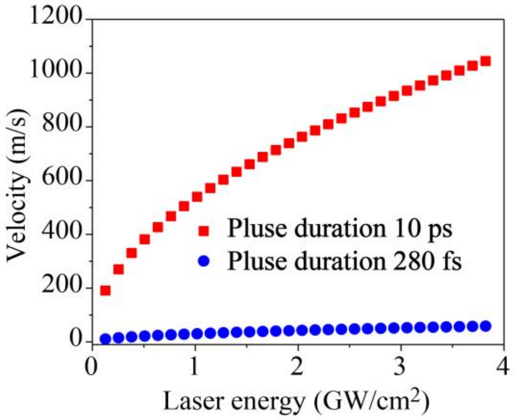

Supplementary Fig.1 Femtosecond laser shock wave velocity. The relationship between the velocity of the shock wave and the laser energy density is calculated.

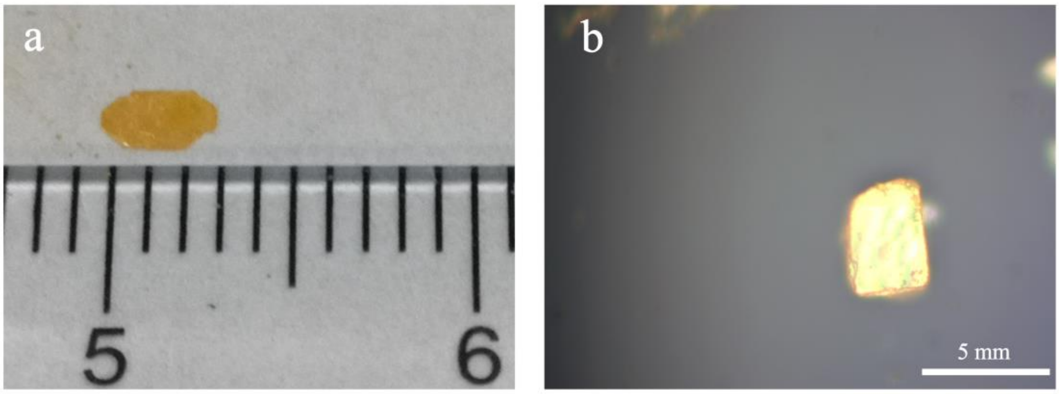

Supplementary Fig.2 Crystal size of (F-PEA)$_2$PbI$_4$ single crystal. a The photograph and **b** microscope.

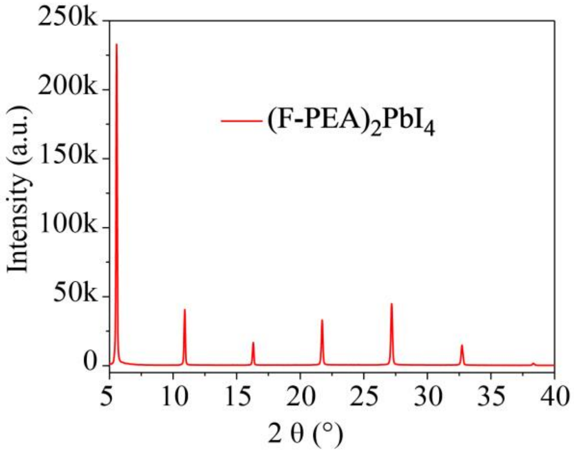

Supplementary Fig.3 The powder X-ray diffraction (PXRD) pattern of (F-PEA)$_2$PbI$_4$ PSCs.

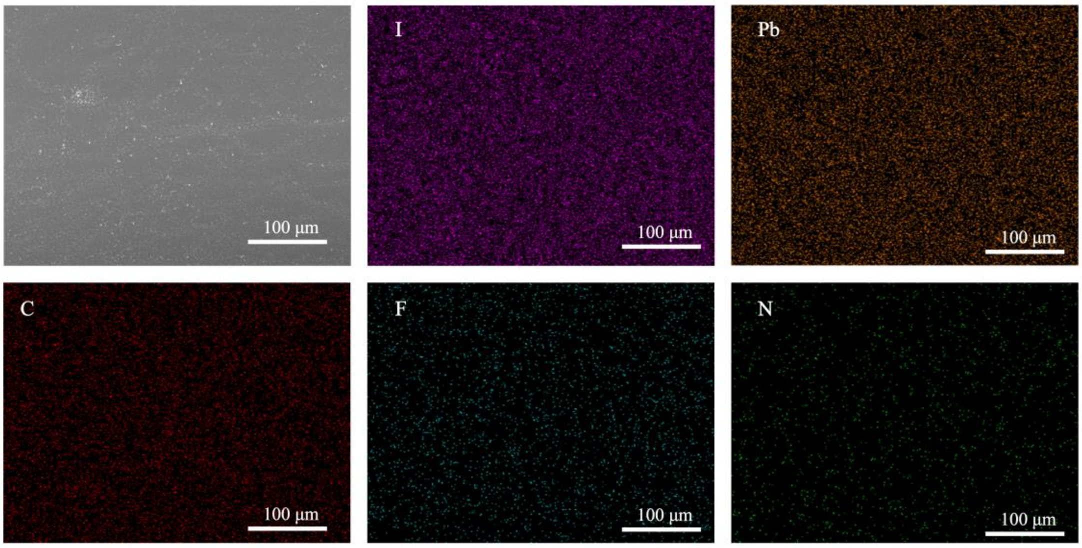

Supplementary Fig.4 Scanning electron microscope (SEM) characterization of (F-PEA)$_2$PbI$_4$ PSCs. The morphology and the corresponding element distribution mapping.

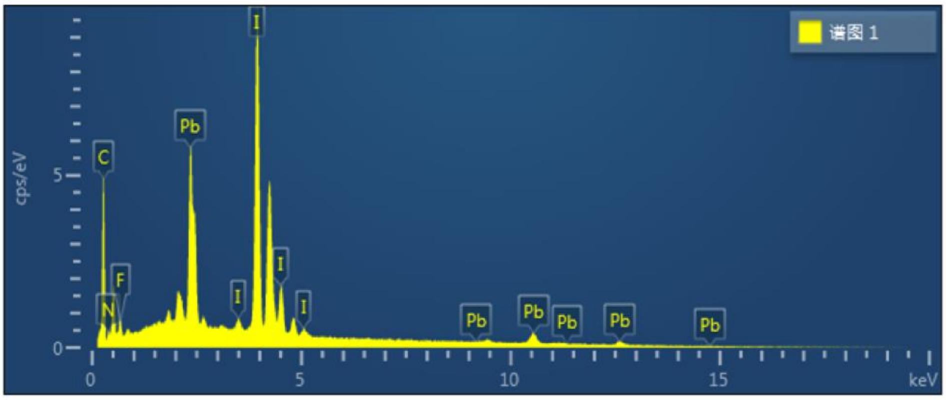

element	Lines type	Apparent concentration	k ratio	wt%	wt% Sigma	Atomic percentage
C	K	5.79	0.05785	19.80	0.29	64.41
N	K	2.33	0.00414	2.79	0.30	7.79
F	K	1.81	0.00356	3.76	0.20	7.72
I	L	31.11	0.31110	52.04	0.35	16.00
Pb	M	14.10	0.13122	21.61	0.30	4.08
Total :				100.00		100.00

Supplementary Fig.5 SEM characterization of (F-PEA)$_2$PbI$_4$ PSCs. The Energy

Disperse Spectroscopy (EDS) concentration and percentage of $(F\text{-}PEA)_2PbI_4$ PSCs.

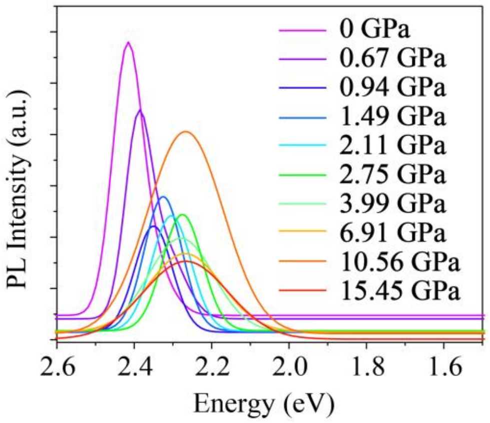

Supplementary Fig.6 Photoluminescence (PL) spectra of $(F\text{-}PEA)_2PbI_4$ PSCs after different laser shocking pressures. We can observe that the PL peak redshift as pressure increased， which demonstrates the Pulsed laser pressure diminution bandgap of perovskite.

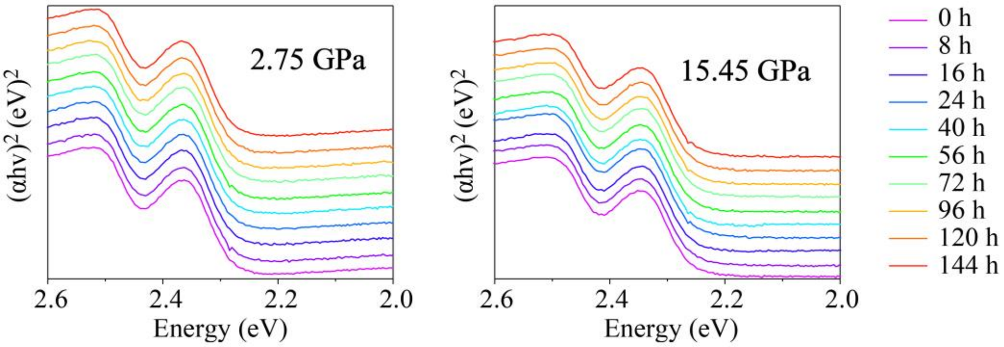

Supplementary Fig.7 Absorption spectrum of $(F\text{-}PEA)_2PbI_4$ PSCs. The bandgap change with time was measured in a room temperature and 85% (±5%) relative humidity ambient atmosphere, completely without encapsulation.

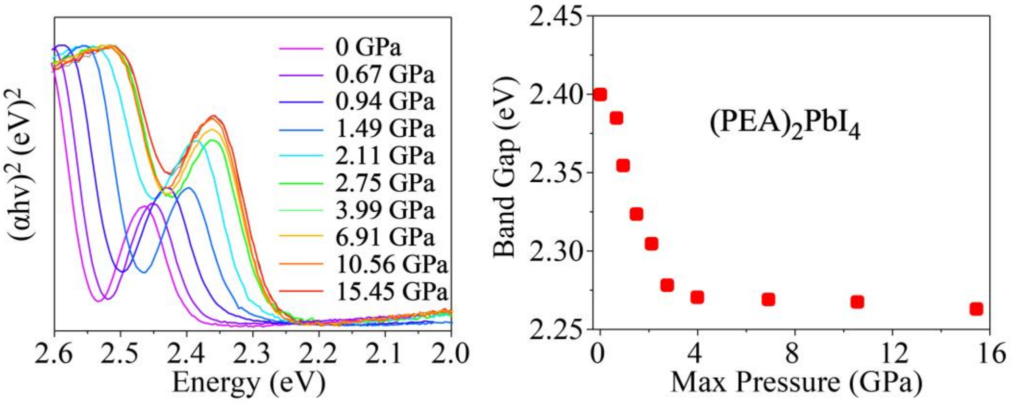

Supplementary Fig.8 Tauc curves obtained from UV–Vis absorption spectra. Absorption spectra reveal bandgap of $(PEA)_2PbI_4$ single crystals variation with ultrafast pressure.

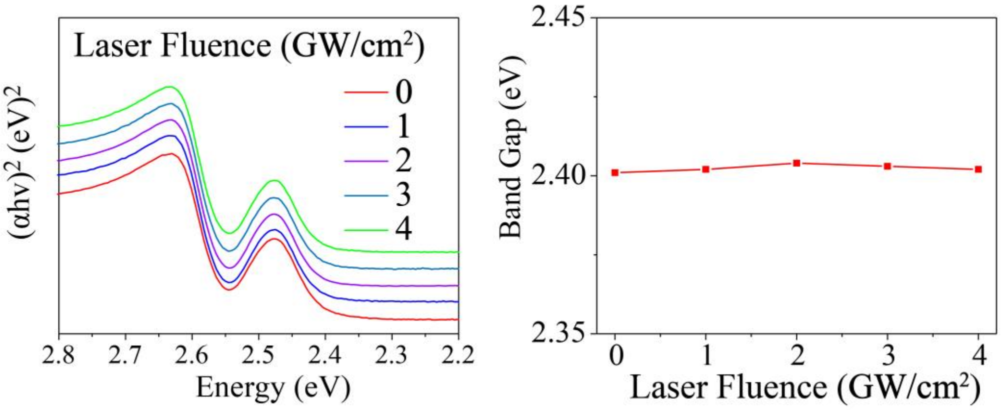

Supplementary Fig.9 Absorption spectrum of $(F\text{-}PEA)_2PbI_4$ PSCs. Under the same conditions as femtosecond laser shock (the structure and wavelength of processing), the $(F\text{-}PEA)_2PbI_4$ PSCs absorption spectrum after being processed by continuous laser scanning. Continuous laser treatment can not regulate the perovskite bandgap.

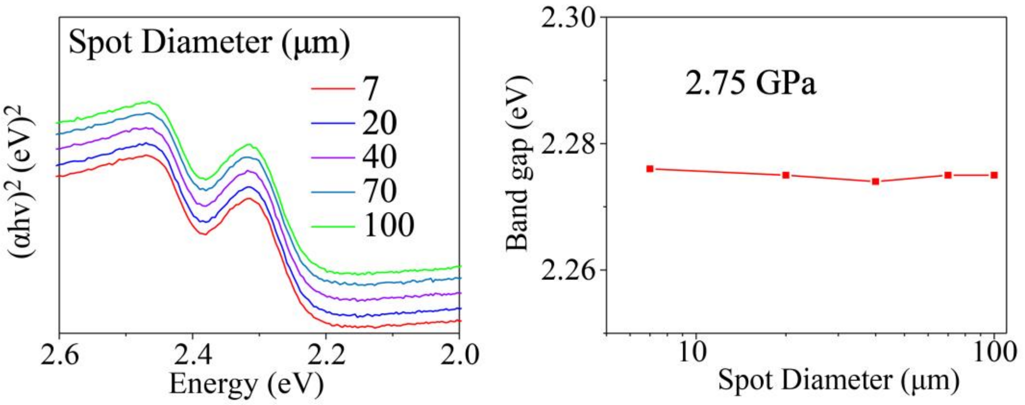

Supplementary Fig.10 Absorption spectrum of $(F\text{-}PEA)_2PbI_4$ PSCs. The change of

(F-PEA)$_2$PbI$_4$ PSCs bandgap with different spot diameters under the same femtosecond laser shock pressure.

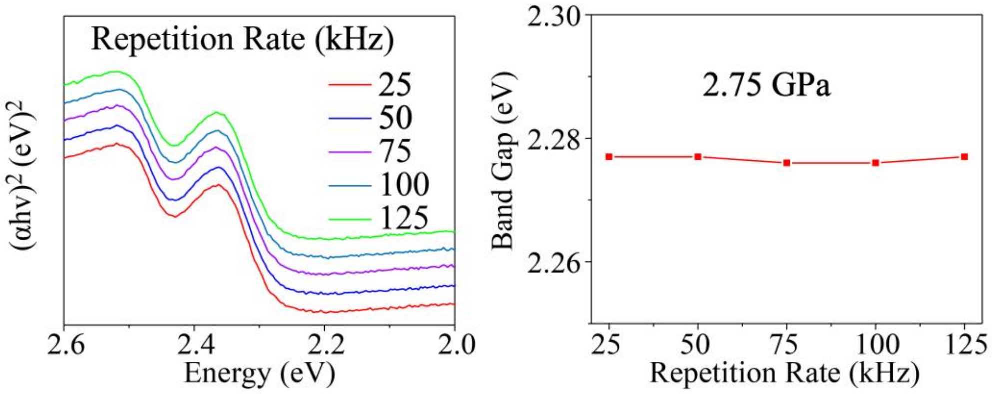

Supplementary Fig.11 Absorption spectrum of (F-PEA)$_2$PbI$_4$ PSCs. The change of (F-PEA)$_2$PbI$_4$ PSCs bandgap with repetition rate under the 2.75 GPa femtosecond laser shock. The modulating of femtosecond laser repetition rate to bandgap can be ignored.

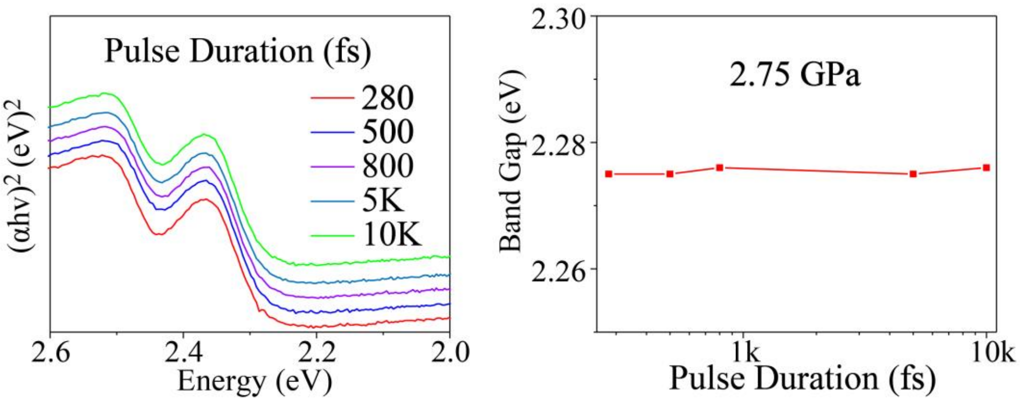

Supplementary Fig.12 Absorption spectrum of (F-PEA)$_2$PbI$_4$ PSCs. (F-PEA)$_2$PbI$_4$ PSCs bandgap as a function of pulse duration at 2.75 GPa.

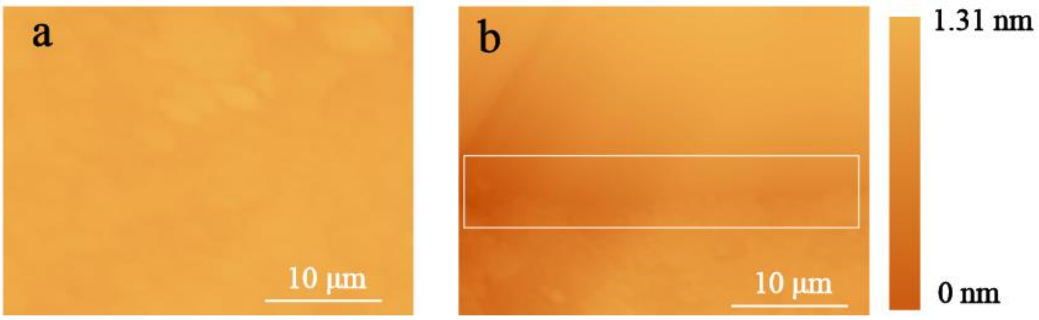

Supplementary Fig.13 Atomic force microscopy (AFM) of (F-PEA)$_2$PbI$_4$ PSCs. (F-PEA)$_2$PbI$_4$ PSCs were linearly scanned at 2.75 GPa, corresponding AFM images **a**

before and **b** after laser shock. The white box represents the femtosecond laser shock area and scanning speed 50 mm/s.

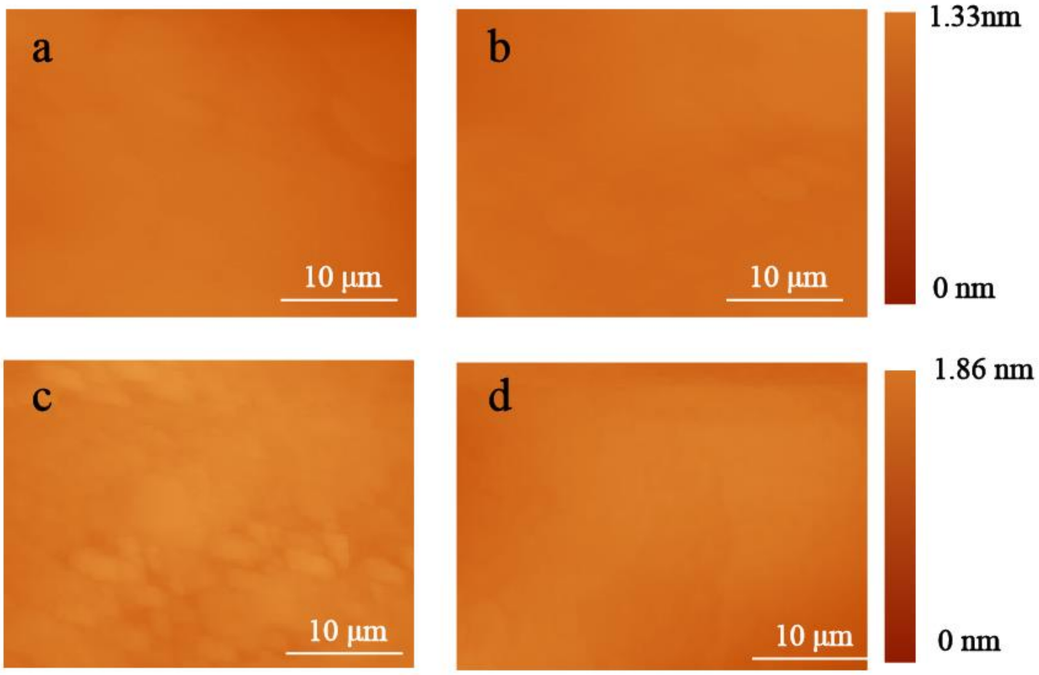

Supplementary Fig.14 Atomic force microscopy (AFM) of (F-PEA)$_2$PbI$_4$ PSCs. (F-PEA)$_2$PbI$_4$ PSCs were scanned at 2.75 GPa, corresponding AFM images **a c** before and **b d** after laser shock. The laser scanning speed 50 mm/s, and the space was **b** 5 and **d** 20 microns apart (spot diameter, 7 μm).

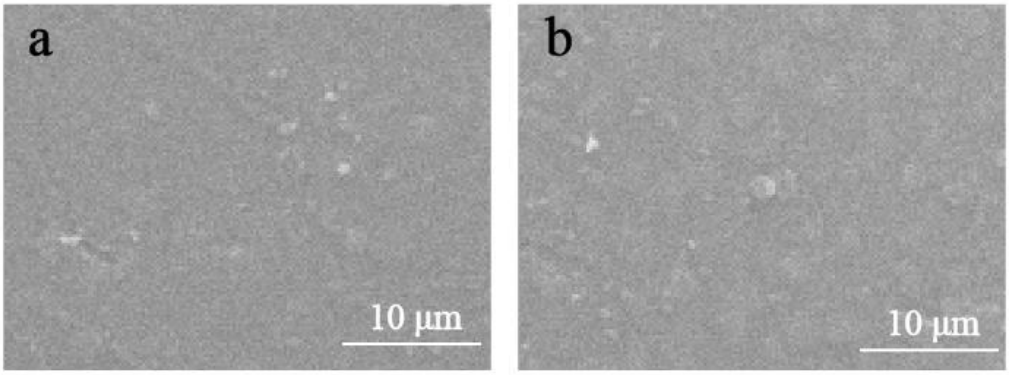

Supplementary Fig.15 SEM images of (F-PEA)$_2$PbI$_4$ PSCs. (F-PEA)$_2$PbI$_4$ PSCs were - scanned at 2.75 GPa, corresponding SEM images **a** before and **b** after laser shock. The laser scans are spaced 20 microns apart and scanning speed 50 mm/s.

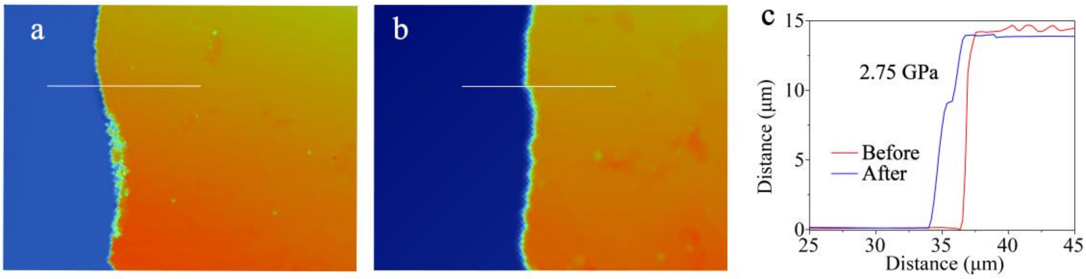

Supplementary Fig.16 Surface profilometer images of (F-PEA)$_2$PbI$_4$ PSCs. The same (F-PEA)$_2$PbI$_4$ PSCs were femtosecond laser shock at 2.75 GPa, corresponding surface profilometer images **a** before and **b** after laser shock. **c** The thickness of the white line. We found that perovskite thickness was reduced by about 0.21 microns by applying an ultra-fast pressure of 2.75 GPa.

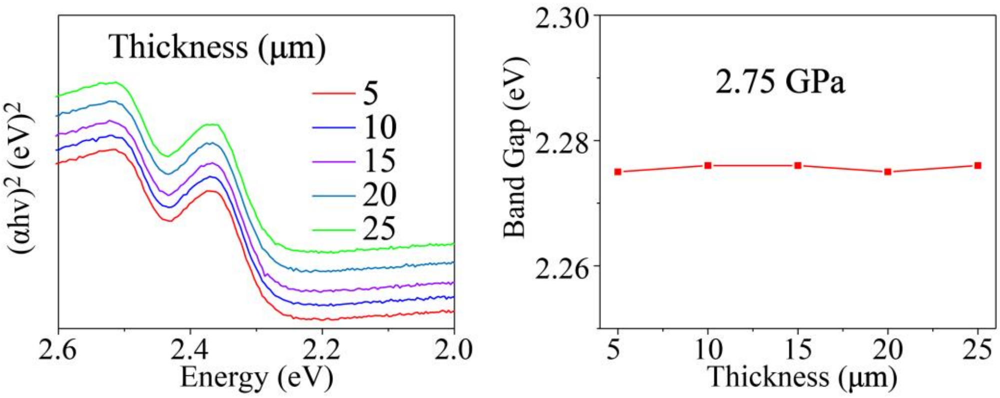

Supplementary Fig.17 Absorption spectrum of (F-PEA)$_2$PbI$_4$ PSCs. The change of (F-PEA)$_2$PbI$_4$ PSCs bandgap with thickness under the 2.75 GPa femtosecond laser shock.

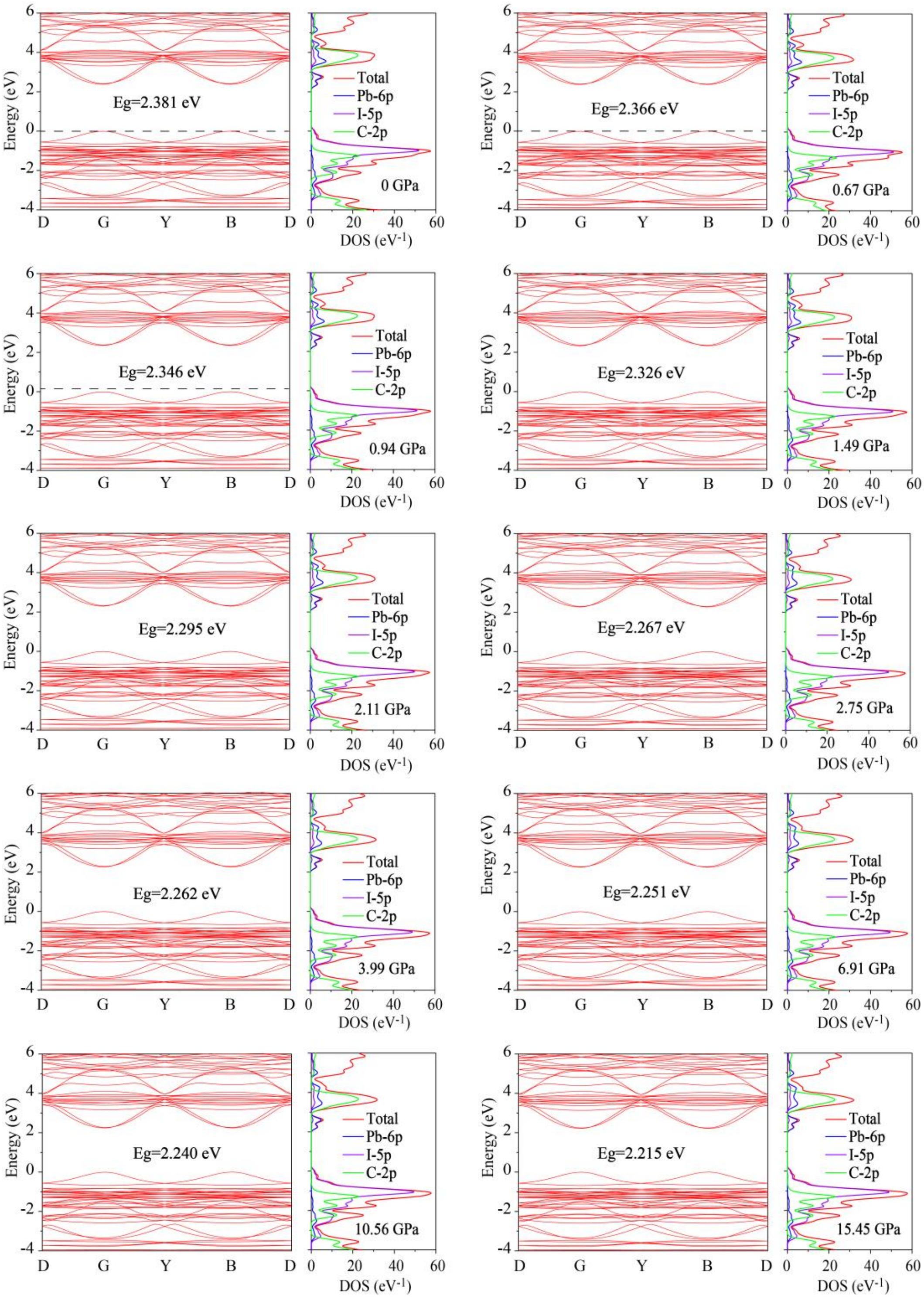

Supplementary Fig.18 Simulation band structure and density of states of (F-PEA)$_2$PbI$_4$ PSCs within various laser shocking pressure. The main contributions of conducting band minimum (CBM) and Valence band maximum (VBM) come from Pb 6*p* and I 5*p*, respectively.

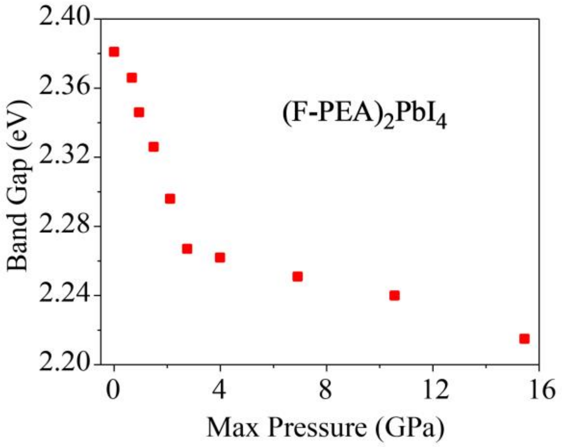

Supplementary Fig.19 Simulated bandgap. The bandgap within various laser shocking pressure of (F-PEA)$_2$PbI$_4$ PSCs.

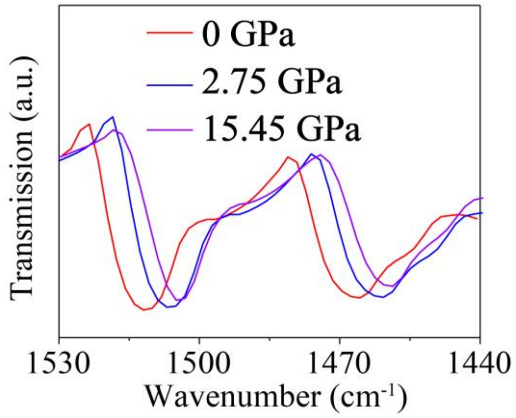

Supplementary Fig.20 Fourier Transform Infrared Spectroscopy (FTIR). FTIR spectra within various laser shocking pressure of (F-PEA)$_2$PbI$_4$ PSCs in the benzene vibration region.

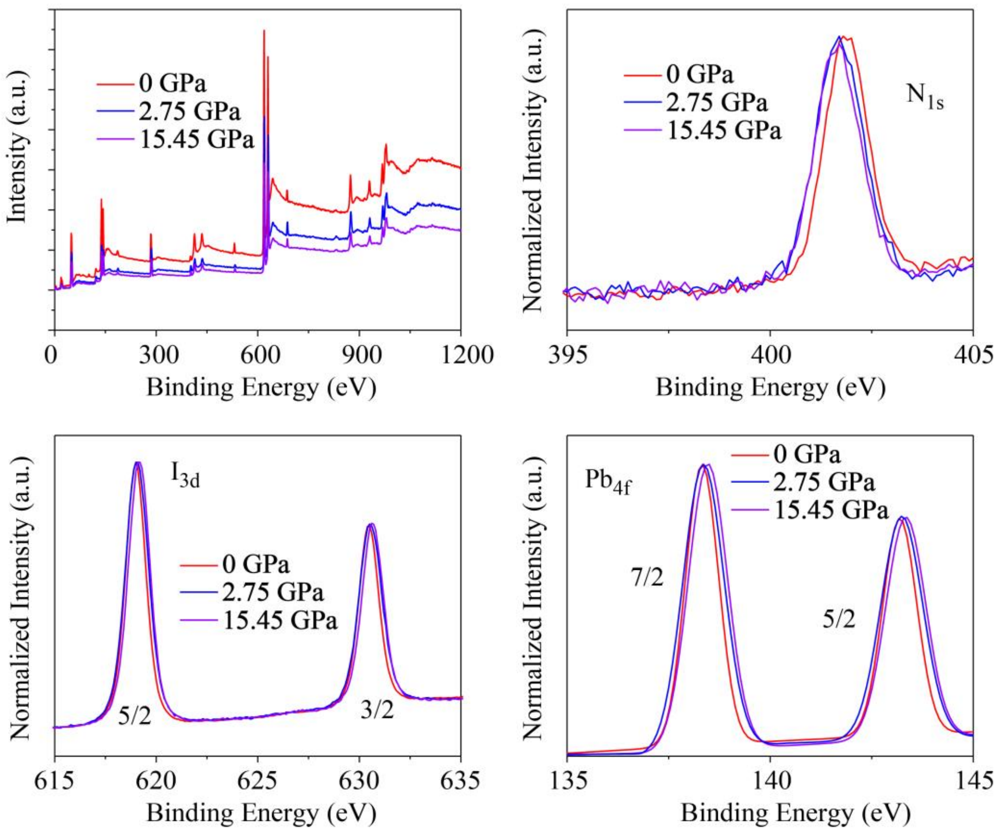

Supplementary Fig.21 X-ray photoelectron spectroscopy (XPS) pattern of (F-PEA)$_2$PbI$_4$ PSCs. Comparison three representative laser shocking pressure, the binding energy is enhanced to some extent.

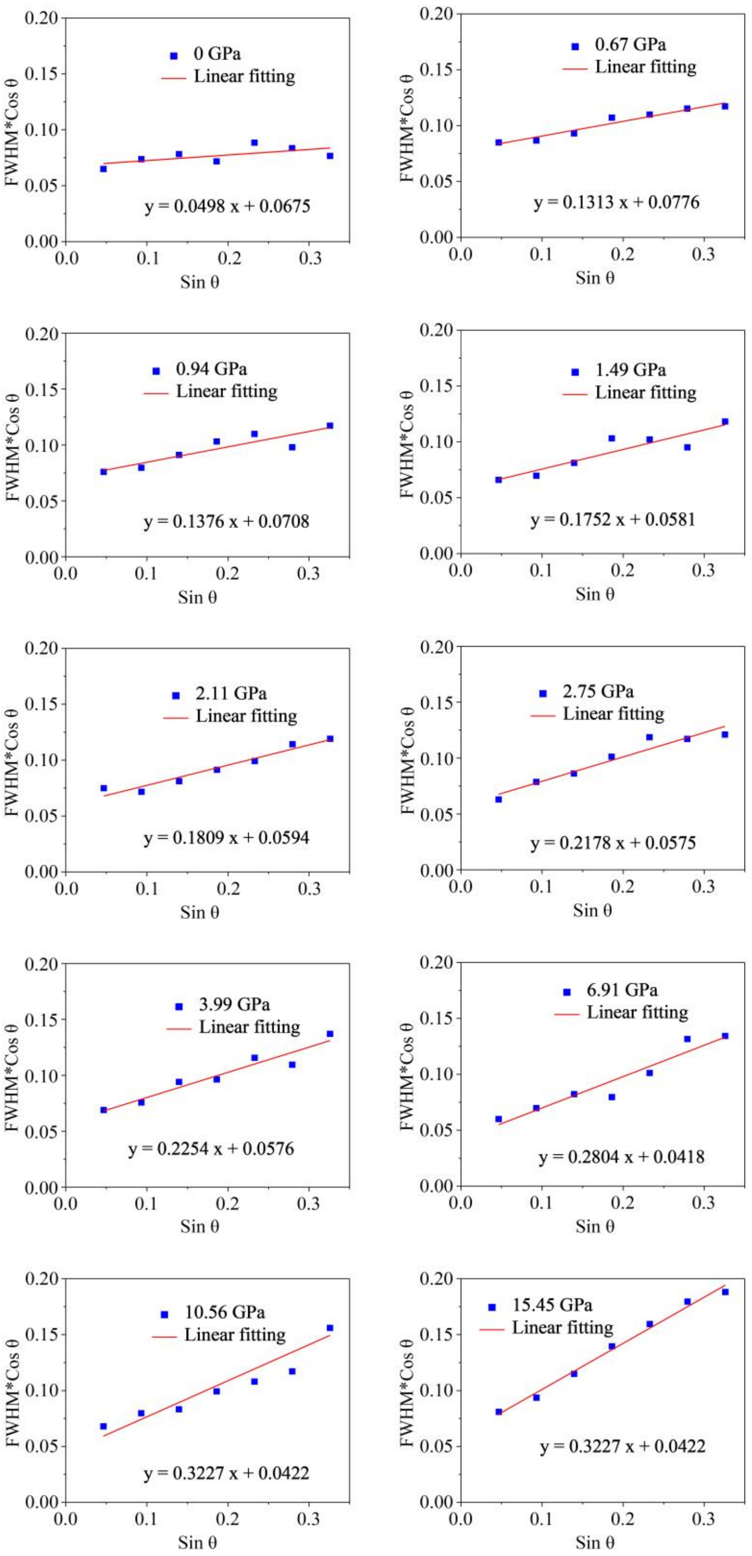

Supplementary Fig.22 Williamson-Hall plot showing the variation of relative strain (slope of the fitted curve, Cε) with different laser shocking pressure. The value of Cε cannot be considered strictly, it is useful for relative meaning.

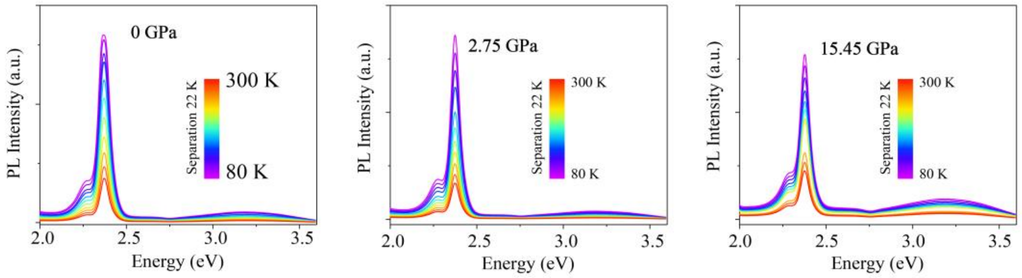

Supplementary Fig.23 Temperature-dependent PL spectra of $(F\text{-}PEA)_2PbI_4$ PSCs.

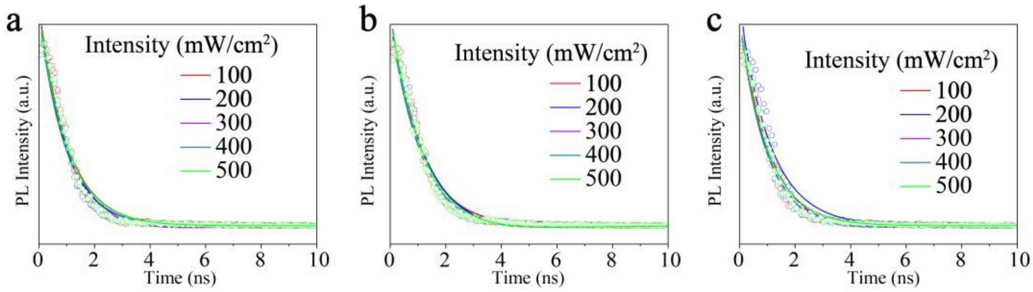

Supplementary Fig.24 Intensity-dependent TRPL spectra of $(F\text{-}PEA)_2PbI_4$ PSCs. **a** 0 GPa, **b** 2.75 GPa and **c** 15.45 GPa ultra-fast pressure.

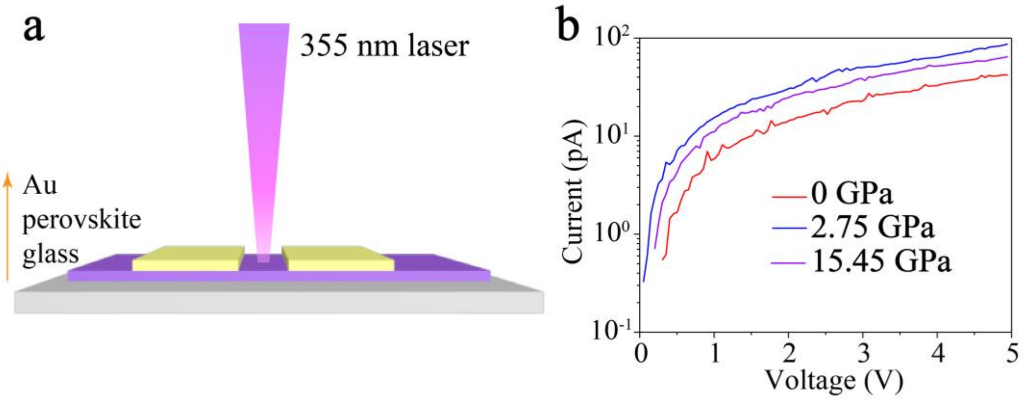

Supplementary Fig.25 Photodetector of $(F\text{-}PEA)_2PbI_4$ PSCs. **a** Schematic diagram of photodetector structure and **b** dark current curve for $(F\text{-}PEA)_2PbI_4$ PSCs.

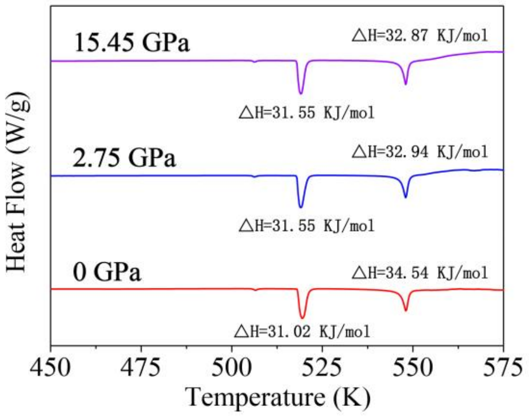

Supplementary Fig.26 Differential Scanning Calorimetry (DSC) pattern of (F-PEA)$_2$PbI$_4$ PSCs in a nitrogen atmosphere. The enthalpy change (ΔH) of the first phase change decreases slightly during the heating, while the enthalpy change of the second phase change decreases slightly.

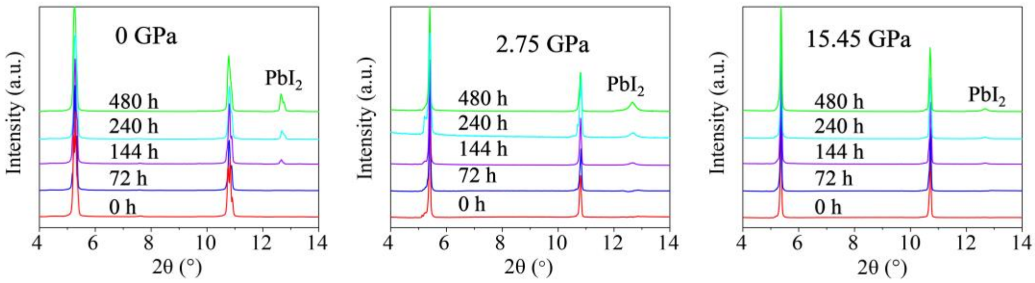

Supplementary Fig.27 Air stability of (F-PEA)$_2$PbI$_4$ PSCs. (F-PEA)$_2$PbI$_4$ PSCs were fabricated and tested in a room temperature and 85% (±5%) relative humidity ambient atmosphere. After 144 hours of testing, perovskite began to decompose into PbI$_2$.

Supplementary Table 1 Crystallographic data for $(F\text{-}PEA)_2PbI_4$ PSCs

Empirical formula	$C_{16}H_{22}N_2F_2PbI_4$
Formula weight	995.14
Crystal system	Monoclinic
Space group	$P2_1/c$ (#14)
Lattice parameters	a= 16.5834(7) Å
	b= 8.5915(4) Å
	c= 8.7609(4) Å
	β= 99.164(2) °
	V= 1232.29(10) $Å^3$
Z value	2
Density (calculated)	2.682 g/cm^3
F(000)	888
Absorption coefficient	36.095 mm^{-1}
Crystal color, habit	Orange, platelet
Crystal dimensions	0.16x0.14x0.015 mm^3

Supplementary Table 2 Atomic coordinates (x10^4) and equivalent isotropic displacement parameters ($Å^2$x10^3) for $(F\text{-}PEA)_2PbI_4$ PSCs. U(eq) is defined as one-third of the trace of the orthogonalized U^{ij} tensor

	x	y	z	U(eq)
C(1)	9145(7)	6371(18)	3608(15)	36(3)
C(2)	8619(7)	3608(15)	4697(13)	28(3)
C(3)	8379(7)	5026(12)	5317(15)	25(3)
C(4)	9101(7)	3587(18)	3544(16)	38(3)
C(5)	9341(8)	4974(13)	3003(17)	29(3)
C(6)	8654(6)	6389(16)	4720(14)	30(3)
C(7)	7866(8)	5066(15)	6539(16)	33(3)
C(8)	6973(8)	4662(15)	5926(15)	30(3)
F(1)	9830(6)	4951(10)	1911(13)	53(3)
I(1)	4999(1)	3127(1)	3107(1)	24(1)
I(2)	3036(1)	168(1)	4290(1)	24(1)
N(1)	6616(6)	5740(11)	4677(10)	24(2)
Pb(1)	5000	0	5000	18(1)

1

Supplementary Table 3 Lattice parameters of $(F\text{-}PEA)_2PbI_4$ PSCs via laser shocking

Max pressure (GPa)	a (Å)	b (Å)	c (Å)	β (°)	V ($Å^3$)
0	16.5835	8.5915	8.7609	99.1642	1232.2910
0.67	16.5534	8.5924	8.7616	99.1641	1230.4563
0.94	16.5070	8.5912	8.7584	99.1645	1226.3881
1.49	16.4609	8.5893	8.7571	99.1598	1222.5259
2.11	16.4150	8.5872	8.7514	99.1642	1218.0135
2.75	16.3542	8.5858	8.7484	99.1658	1212.8864
3.99	16.3391	8.5853	8.7482	99.1661	1211.6660
6.91	16.3240	8.5847	8.7460	99.1667	1210.1559
10.56	16.3090	8.5841	8.7452	99.1672	1208.8425
15.45	16.2640	8.5832	8.7443	99.1681	1205.2522

Supplementary Table 4 Bond lengths and angles for (F-PEA)$_2$PbI$_4$ PSCs via laser shocking.

Max pressure（GPa）	Pb-I axial（Å）	Pb-I equatorial（Å）	I-Pb-I axial（°）	I-Pb-I equatorial（°）	Pb-I-Pb（°）
0	3.219	3.163	179.508	88.903	152.267
0.67	3.214	3.163	179.474	88.905	152.265
0.94	3.205	3.162	179.434	88.916	152.263
1.49	3.196	3.161	179.390	88.913	152.262
2.11	3.187	3.159	179.356	88.933	152.261
2.75	3.176	3.158	179.302	88.943	152.260
3.99	3.173	3.158	179.286	88.941	152.260
6.91	3.170	3.158	179.276	88.950	152.259
10.56	3.167	3.157	179.262	88.951	152.259
15.45	3.158	3.157	179.218	88.951	152.258

Supplementary Table 5 Summary energy levels of (F-PEA)$_2$PbI$_4$ PSCs obtained from UPS

Max pressure（GPa）	Work function (eV)	VBM (eV)	Eg (eV)	CBM (eV)
0	5.33	-6.47	2.41	-4.06
2.75	5.44	-6.53	2.28	-4.25
15.45	-4.25	-6.61	2.26	-4.35

Supplementary Table 6 Summary of (F-PEA)$_2$PbI$_4$ PSCs lifetime at 0 GPa

Excitation Intensity (mW/cm^2)	τ_1 (ns)	τ_2 (ns)	τ (ns)
100	0.80	3.55	1.20
200	0.79	3.18	1.03
300	0.76	2.72	1.06
400	0.77	2.80	1.07

500	0.80	3.06	1.14

Supplementary Table 7 Summary of $(F\text{-}PEA)_2PbI_4$ PSCs lifetime at 2.75 GPa

Excitation Intensity (mW/cm^2)	τ_1 (ns)	τ_2 (ns)	τ (ns)
100	0.98	7.79	2.21
200	0.98	7.42	2.38
300	0.96	7.24	2.25
400	0.99	6.86	2.24
500	1.01	7.22	2.12

Supplementary Table 8 Summary of $(F\text{-}PEA)_2PbI_4$ PSCs lifetime at 15.45 GPa

Excitation Intensity (mW/cm^2)	τ_1 (ns)	τ_2 (ns)	τ (ns)
100	0.93	3.72	1.32
200	0.88	2.35	1.21
300	0.88	2.82	1.13
400	0.93	2.96	1.19
500	0.96	2.83	1.26
